# Effectiveness of Ultrasound-Guided Stellate Ganglion Block in Treating Pediatric Complex Regional Pain Syndrome

**DOI:** 10.7759/cureus.77002

**Published:** 2025-01-06

**Authors:** Kevin M Tang, Crystal Lee, Mia Castiglione, Haijun Zhang

**Affiliations:** 1 Anesthesiology and Perioperative Medicine, Rutgers University New Jersey Medical School, Newark, USA; 2 Anesthesiology, Rutgers University New Jersey Medical School, Newark, USA

**Keywords:** complex regional pain syndrome type 1, pain intervention, pediatric pain, stellate ganglion block (sgb), ultrasound-guided

## Abstract

Complex regional pain syndrome (CRPS) is a painful neuropathic condition that is often difficult to diagnose and treat in children. In patients who fail conservative treatment, an interventional approach can provide pain relief and improve functionality. We present a case in which an ultrasound-guided stellate ganglion block (SGB) was performed on a pediatric patient with refractory CRPS affecting the upper extremity. Following the block, the patient reported significant pain reduction, less frequent flares, and improvement of functions without any significant complications. This case report aims to highlight the effectiveness of ultrasound-guided SGB in treating pediatric CRPS patients. The recent advocacy of ultrasound-guided interventions has demonstrated efficacy and improved safety due to clear visualization of key structures.

## Introduction

Complex regional pain syndrome (CRPS) is a debilitating condition that is often difficult to diagnose in children. This condition is characterized by chronic, spontaneous, and provoked pain in the distal extremities, which can lead to chronic psychosocial dysfunction in children and adolescents [1,2]. The specific causes of CRPS remain unclear. However, CRPS often follows minor injuries such as sprains, dislocations, or soft tissue damage [3,4]. Several studies have also noted that stress plays a pivotal role in triggering or perpetuating CRPS. It has been observed that pediatric CRPS patients are prone to anxiety and other forms of heightened somatic symptoms and emotional distress [5].

Clinical observations in pediatric CRPS patients reveal a spectrum of sensory and motor abnormalities. Key features include heightened sensitivity to painful and non-painful stimuli in the affected limb, along with autonomic dysfunction, motor impairments, and trophic changes from nerve supply disruption [3,6]. Pediatric patients typically report chronic pain in the affected limb, even during rest, which is exacerbated with limb movement [6,7]. While physical therapy, occupational therapy, and cognitive behavioral therapy are recognized as first-line treatments for pediatric CRPS, patients who are refractory to treatment may require further pharmacological or interventional management to achieve appropriate pain relief [8]. We present a case in which an ultrasound-guided SGB was performed to significantly relieve pain in a pediatric patient with refractory CRPS affecting the upper extremity.

## Case presentation

A 15-year-old otherwise healthy female presented with a one-year history of 8/10 continuously pulsating and stabbing left wrist and left forearm pain that occasionally radiates into her left dorsum index, thumb, and middle finger. The patient reported significant pain with any movement of her left arm and an inability to participate in physical activities at school. Prior to her arrival at the pain clinic, the patient received X-rays of her left arm, which showed no acute fracture, and an MRI of her left extremity, which showed swelling in the dorsal intercarpal ligament. She was then referred to an orthopedic hand surgeon who initiated physical and occupational therapy, nonsteroidal anti-inflammatory drug (NSAID) therapy, and wrist splinting. Upon completing those treatments, the patient did not experience any pain relief. Initial workup, including repeat MRI and X-ray of the left forearm, showed normal soft tissue without any acute fractures or inflammatory changes. Her physical exam was notable for allodynia on the dorsal and dorsolateral aspect of her left forearm and wrist with slight skin color changes. The diagnosis of CRPS type I was made using the Budapest criteria.

**Figure 1:**
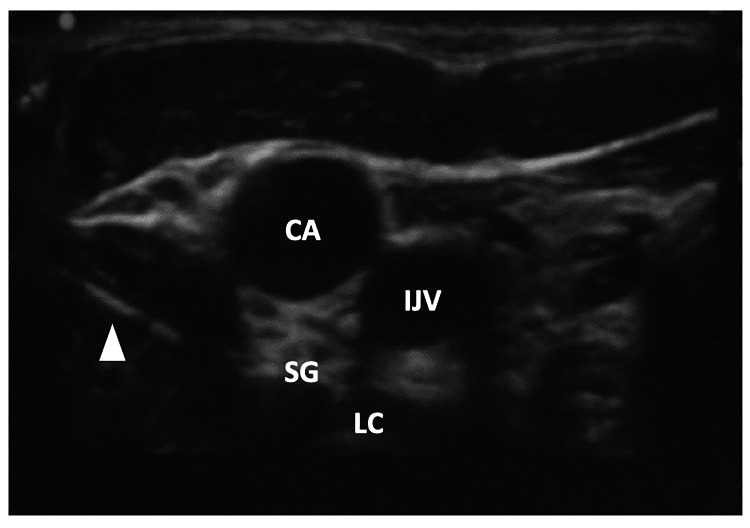
Ultrasound image shows key anatomical structures during stellate ganglion block (SGB). The needle is indicated by the arrowhead. CA: carotid artery; IJV: internal jugular vein; SG: stellate ganglion; LC: longus colli muscle

Given the patient’s lack of response to conservative medical management, a left SGB under ultrasound guidance was performed. Briefly, the patient was placed supine with the head slightly turned to the contralateral side. An ultrasound probe was used to visualize and identify C6 and C7 cervical articular pillars. The probe was then transitioned anteriorly to visualize vital anatomy, including the internal jugular vein, carotid artery, thyroid, thyroid artery, and longus colli muscle (Figure 1). With an in-plane approach, a spinal needle was advanced above the longus colli muscle, and after negative aspiration for blood, fluid, or air, a mixture of 5 ml 0.25% bupivacaine and 40 mg (1 ml) triamcinolone was injected. The patient tolerated the procedure well and reported no new neurological deficits during subsequent clinic visits.

Following her initial SGB, the patient reported 100% pain relief immediately post-procedure, and at her four-week follow-up, she reported improvement of pain to 5/10 and the ability to reach over her head without discomfort. During this time, the patient was prescribed 30 mg oral duloxetine once at night, which further improved her symptoms. Three months after her initial SGB, the patient required a repeat injection, which again significantly relieved her pain and functional status for six months. The patient then received the third SGB and never required more injections. The patient is now able to tolerate physical activity and can use her left extremity in her activities of daily living without significant pain.

## Discussion

The management of children with CRPS can be very challenging due to the high physical burden of intensive physical therapy, increased risk of somatic and psychological distress, and lack of large-scale pediatric-focused studies [9,10]. In a Canadian surveillance study conducted by Baerg et al., the incidence of CRPS in children was 1.14 in 100,000, with the mean age of diagnosis being 12.2 years old. Of those being medically treated, 82.7% and 66.0% were treated with NSAIDs and acetaminophen, respectively [11]. In a network meta-analysis conducted by Wertli et al., conventional analgesics for CRPS (paracetamol, NSAIDs, and opioids) have shown minimal to no statistically significant improvement in long-term pain outcomes in adults [12]. Similarly, Breuer et al. concluded in their randomized, placebo-controlled, double-blind trial that short-term treatment with the selective COX-2 inhibitor parecoxib showed no significant change in pain scores in patients with CRPS type I [13]. In more recent years, the use of bisphosphonates, gabapentin, ketamine, and antiepileptics has been under investigation in small-scale clinical trials; however, the efficacy of such treatments is limited and inconsistent [14].

In this case, we report the use of ultrasound-guided SGB for a pediatric patient with CRPS who experienced significant pain relief with functional improvement. The efficacy of invasive intervention is poorly understood in pediatric CRPS. In a scoping review, the difficulty of diagnosing CRPS due to the broad differential diagnosis for pediatric chronic pain prevents conclusions on treatment effectiveness from being drawn [8]. So far, only one placebo-controlled crossover trial has studied the effectiveness of lumbar sympathetic blockade in children with CRPS. In this study, 23 patients, aged 10-18 years old, reported a significant reduction in pain intensity comparing lumbar sympathetic blockade with lidocaine to intravenous lidocaine [15].

It has previously shown that SGB under ultrasound guidance can mitigate the risk of block failure due to incorrect fascial plane identification and improve visualization of key structures, including the esophagus, vertebral arteries, carotid vessels, thyroid gland, and thyroid vessels [16]. While few randomized controlled trials have been done to evaluate the safety profile of ultrasound-guided versus fluoroscope-guided SGB, several observational studies have demonstrated that ultrasound guidance provides a distinctive advantage in soft tissue visualization and lack of radiation exposure that may be beneficial for pediatric patients [17,18]. This claim is further substantiated by Imani et. al., who showed significant disability improvement and decreased reported unpleasant effects post-procedure in patients after ultrasound-guided SBG compared to fluoroscopic guidance [19].

Ultrasound has several distinctive advantages compared to conventional fluoroscopic imaging in that both the patient and the provider are not exposed to ionizing radiation, the equipment size and cost are reduced, and key vasculature and nerve structures are better visualized. While this adds to the small but growing literature on the interventional management of pediatric CRPS, we hope to encourage future researchers to investigate the long-term pain outcomes and intraoperative complication rates of ultrasound versus fluoroscopy-guided SGB in children.

## Conclusions

It is challenging to diagnose and treat pediatric CRPS. Ultrasound-guided SGB can be performed effectively to relieve pain and restore functions in pediatric CRPS patients. The use of ultrasound guidance can mitigate the risk of block failure due to more accurate facial plane identification and improved visualization of key structures. Other benefits of using ultrasound guidance include avoidance of exposure to radiation, easy access to the portable device, and reduced size and cost of the equipment.

## References

[1] Vescio A, Testa G, Culmone A, Sapienza M, Valenti F, Di Maria F, Pavone V (2020). Treatment of complex regional pain syndrome in children and adolescents: a structured literature scoping review. Children (Basel).

[2] Logan DE, Williams SE, Carullo VP, Claar RL, Bruehl S, Berde CB (2013). Children and adolescents with complex regional pain syndrome: more psychologically distressed than other children in pain?. Pain Res Manag.

[3] Low AK, Ward K, Wines AP (2007). Pediatric complex regional pain syndrome. J Pediatr Orthop.

[4] Ho ES, Ponnuthurai J, Clarke HM (2014). The incidence of idiopathic musculoskeletal pain in children with upper extremity injuries. J Hand Ther.

[5] Cruz N, O'Reilly J, Slomine BS, Salorio CF (2011). Emotional and neuropsychological profiles of children with complex regional pain syndrome type-I in an inpatient rehabilitation setting. Clin J Pain.

[6] Sethna NF, Meier PM, Zurakowski D, Berde CB (2007). Cutaneous sensory abnormalities in children and adolescents with complex regional pain syndromes. Pain.

[7] Borucki AN, Greco CD (2015). An update on complex regional pain syndromes in children and adolescents. Curr Opin Pediatr.

[8] Zernikow B, Wager J, Brehmer H, Hirschfeld G, Maier C (2015). Invasive treatments for complex regional pain syndrome in children and adolescents: a scoping review. Anesthesiology.

[9] Sherry DD, Wallace CA, Kelley C, Kidder M, Sapp L (1999). Short- and long-term outcomes of children with complex regional pain syndrome type I treated with exercise therapy. Clin J Pain.

[10] Katholi BR, Daghstani SS, Banez GA, Brady KK (2014). Noninvasive treatments for pediatric complex regional pain syndrome: a focused review. PM R.

[11] Baerg K, Tupper SM, Chu LM (2022). Canadian surveillance study of complex regional pain syndrome in children. Pain.

[12] Wertli MM, Kessels AG, Perez RS, Bachmann LM, Brunner F (2014). Rational pain management in complex regional pain syndrome 1 (CRPS 1) - a network meta-analysis. Pain Med.

[13] Breuer AJ, Mainka T, Hansel N (2014). Short-term treatment with parecoxib for complex regional pain syndrome: a randomized, placebo-controlled double-blind trial. Pain.

[14] Weissmann R, Uziel Y (2016). Pediatric complex regional pain syndrome: a review. Pediatr Rheumatol Online J.

[15] Meier PM, Zurakowski D, Berde CB, Sethna NF (2009). Lumbar sympathetic blockade in children with complex regional pain syndromes: a double blind placebo-controlled crossover trial. Anesthesiology.

[16] Narouze S (2014). Ultrasound-guided stellate ganglion block: safety and efficacy. Curr Pain Headache Rep.

[17] Siegenthaler A, Mlekusch S, Schliessbach J, Curatolo M, Eichenberger U (2012). Ultrasound imaging to estimate risk of esophageal and vascular puncture after conventional stellate ganglion block. Regional anesthesia and pain medicine.

[18] Bhatia A, Flamer D, Peng PW (2012). Evaluation of sonoanatomy relevant to performing stellate ganglion blocks using anterior and lateral simulated approaches: an observational study. Can J Anaesth.

[19] Imani F, Hemati K, Rahimzadeh P, Kazemi MR, Hejazian K (2016). Effectiveness of stellate ganglion block under fuoroscopy or ultrasound guidance in upper extremity CRPS. J Clin Diagn Res.

